# Association between neutrophil percentage-to-albumin ratio and all-cause mortality in stroke survivors stratified by triglyceride-glucose index: A prospective observational study based on the NHANES database from 1999 to 2018

**DOI:** 10.1097/MD.0000000000050968

**Published:** 2026-10-09

**Authors:** Ning Liu, Zhongzhou Su, Sheng Zhang

**Affiliations:** a Department of Neurosurgery, Huzhou Central Hospital, Fifth School of Clinical Medicine of Zhejiang Chinese Medical University, Huzhou, Zhejiang, China.

**Keywords:** all-cause mortality, NPAR, stroke, survivor, TyG

## Abstract

The neutrophil percentage-to-albumin ratio (NPAR) is a novel inflammatory biomarker with prognostic value in critical illnesses, but its association with outcomes in stroke survivors remains unclear. No prior study has explored whether this association differs across triglyceride-glucose index (TyG) strata, a surrogate marker of insulin resistance (IR) and metabolic disturbance in cardiovascular disease. Data from the National Health and Nutrition Examination Survey 1999 to 2018 were analyzed for 1187 stroke survivors aged 40 to 80 years. Kaplan–Meier curves, multivariate Cox models, restricted cubic spline (RCS) analysis, and subgroup analyses were used to examine the relationship between baseline NPAR and all-cause mortality across TyG levels. Over a median follow-up of 86 months, 496 (41.79%) of the 1187 participants died; median survival was 133 months. Kaplan–Meier analysis showed significant mortality differences across NPAR tertiles in all participants and within intermediate- and high-IR groups (all *P* < .001). As a continuous variable, NPAR was significantly associated with mortality in the intermediate-risk (hazard ratio = 1.18) and high-risk (hazard ratio = 1.10) groups, but not in the low-risk group. As a categorical variable, the highest NPAR tertile (T3) was associated with higher mortality across all adjustment models in IR intermediate- and high-risk groups, whereas in the IR low-risk group, significance was observed only in the minimally adjusted model. Restricted cubic spline analysis revealed a linear association between NPAR and mortality in all participants (*P* for nonlinear = .291), as well as in IR intermediate- and high-risk groups, but no association in the IR low-risk group (*P* for overall = .187). Subgroup analyses showed significant interactions across TyG subgroups (*P* for interaction = .047). Two sensitivity analyses confirmed the stability of the main findings. Monitoring NPAR levels alongside TyG-based stratification may facilitate comprehensive risk assessment and inform targeted management strategies for stroke survivors.

## 1. Introduction

Stroke represents a major global health burden, ranking as the 2nd leading cause of mortality worldwide and the primary contributor to long‑term disability. Particularly concerning is its escalating incidence in developing nations, where healthcare systems face growing challenges in stroke prevention and management.^[1]^ Although stroke mortality has decreased globally in recent decades, the decline in stroke incidence has decelerated, and the global burden of the disease remains considerable.^[2]^ At the same time, understanding the predictors of stroke outcomes has become an area of intense research focus. The neutrophil percentage-to-albumin ratio (NPAR) is a recently identified inflammatory biomarker that demonstrates predictive and prognostic value in a range of critical illnesses.^[3,4]^ However, existing research has predominantly focused on the acute phase of stroke, employing logistic regression models to investigate its impact on short-term mortality or 3-month functional outcomes. For stroke survivors who have progressed beyond the acute phase, chronic low-grade inflammation may exert a more profound influence on their long-term survival. Currently, there is a paucity of studies utilizing Cox regression models based on extended follow-up to explore the association between NPAR and long-term mortality in this population. Notably, insulin resistance (IR), a core component of metabolic syndrome, often exhibits a bidirectional relationship with inflammatory states. Under conditions of high IR, the inflammatory response may be amplified, potentially modifying the association between NPAR and mortality risk. This suggests that the prognostic value of NPAR may vary among stroke survivors with different metabolic profiles. Therefore, this study aims to analyze the association between NPAR and all-cause mortality using data from stroke survivors in the National Health and Nutrition Examination Survey (NHANES) database from 1999 to 2018 and to further explore whether IR modifies this relationship. We hypothesize that elevated NPAR is independently associated with an increased risk of long-term mortality in stroke survivors, and that this association is more pronounced in individuals with IR. These findings may generate hypotheses regarding a novel inflammatory biomarker for long‑term risk stratification among stroke survivors, and provide epidemiological evidence supporting potential personalized anti‑inflammatory or nutritional intervention strategies tailored to distinct metabolic phenotypes.

## 2. Methods

### 2.1. Study population and ethics

This study is a prospective cohort observational study that utilized participants from the NHANES database from 1999 to 2018. Inclusion criteria: Participants were eligible if they: had a prior diagnosis of stroke and were aged 40 to 80 years. Exclusion criteria: pregnancy; patients with liver disease or cancer; neutrophil percentage, albumin, triglyceride (TG), and fasting plasma glucose (FPG) were missing. A total of 1187 participants were included according to the inclusion and exclusion criteria. The detailed flow chart of participant recruitment is shown in Figure 1.

**Figure 1.**
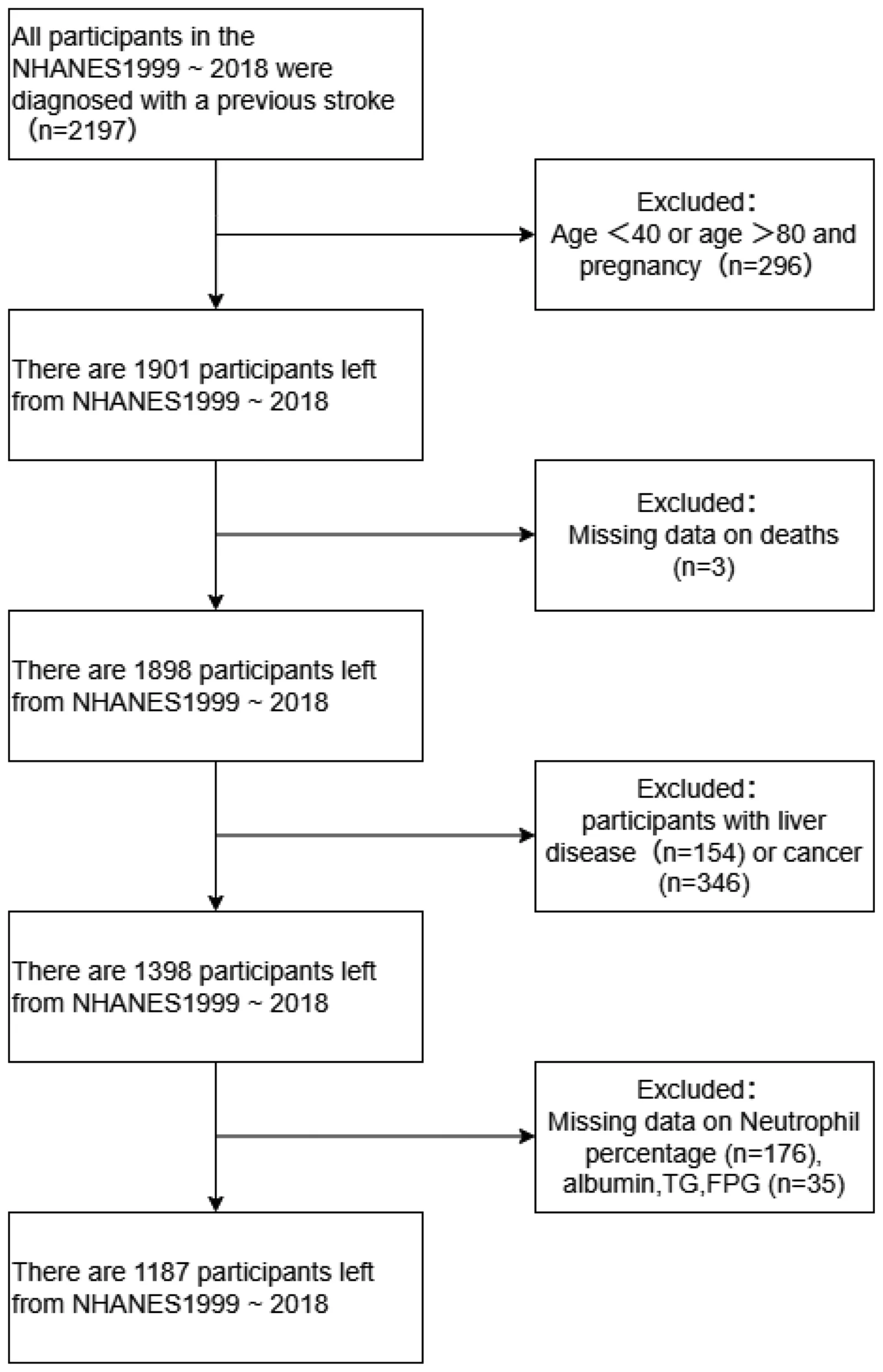
The flowchart of study participants. FPG = fasting plasma glucose, NHANES = National Health and Nutrition Examination Survey, TG = triglycerides.

### 2.2. Data collection

Demographic data provided information on gender, age, race, marital status, education level, family income-to-poverty ratio, and pregnancy status. Body measurements were used to obtain height and weight. Self-reported questionnaire data included medical history of stroke, liver disease, cancer, hypertension, diabetes (or prediabetes), kidney disease, chronic bronchitis, and smoking status. Laboratory analyses yielded the following fasting plasma biomarkers: TG, albumin, FPG, glycosylated hemoglobin (HbA1c), neutrophil percentage, white blood cell count (WBC), hemoglobin (Hb), and platelet count.

### 2.3. Definitions

Gender, age, race, marital status, education level, ratio of family income to poverty, and pregnancy, smoking through self-assessment.

History of stroke, liver disease, cancer, hypertension, coronary heart disease, kidney disease, and chronic bronchitis were assessed by self-reported physician diagnoses.

The NPAR was calculated as the percentage of neutrophils (%)/albumin (g/dL).

Body mass index (BMI) was calculated using the formula: BMI = weight (kg)/height (m)^2^, and height, weight were collected by trained health technicians at mobile examination centers.

The glucose metabolism level was divided into diabetes mellitus (DM) group, pre-DM group, and normal glucose regulation group. The DM group was defined as FPG ≥ 7.0 mmol/L or HbA1c ≥ 6.5%, or self-reported physician diagnosis, and/or use of antidiabetic drugs. The pre-DM group was defined as 6.1 ≤ FPG <7 mmol/L, or 5.7% < HbA1c <6.4%, or a self-reported physician’s diagnosis. Those who did not meet the diagnosis of DM group and pre-DM group were classified as normal glucose regulation group.

IR was assessed by the triglyceride-glucose index (TyG). TyG = ln[TG (mg/dL) × FPG (mg/dL)/2]. According to the level of TyG, the patients were divided into a low-risk group (TyG < 8.5), an intermediate-risk group (8.5 ≤ TyG ≤ 9.0), and a high-risk group (TyG > 9.0).

### 2.4. Follow-up of endpoint events

We determined mortality status in the follow-up population using the NHANES public-use mortality file through December 31, 2019, which was linked to the National Death Index via probabilistic matching. The follow-up period was calculated as the number of months from the interview date to either the date of death (for deceased participants) or December 31, 2019 (for surviving participants).

### 2.5. Screening of covariates

Covariate selection was performed using a multi-step approach. First, univariate Cox regression analyses were conducted for preliminary screening, and variables with *P* < .10 were retained as candidate predictors for least absolute shrinkage and selection operator (LASSO) regression. Because LASSO regression may identify only a limited number of predictors, elastic-net regression was subsequently applied to expand the selection of candidate covariates. Furthermore, Hb and BMI were a priori forced into the covariate set, given prior evidence of their predictive value for mortality in stroke patients.^[5–8]^

### 2.6. Statistical analysis

The R (version 4.3.2; R Core Team, Vienna, Austria) statistical analysis software was used in this study. Continuous data with normal distribution were expressed as mean ± standard deviation, and 1-way analysis of variance was used to analyze statistical significance. Continuous data with non-normal distribution were presented as median and interquartile range and analyzed by the Kruskal–Wallis test. Categorical data were described with counts and percentages and assessed using the chi-square test. Participants were stratified into tertiles based on NPAR levels: *T*1 (NPAR ≤ 13.5), *T*2 (13.5 < NPAR ≤ 15.7), and *T*3 (NPAR > 15.7). To ensure robust findings, NPAR was analyzed both as a categorical and continuous variable. Stroke survivor mortality rates were estimated using Kaplan–Meier (KM) curves, with between-group differences assessed via the log-rank test. Univariate Cox regression was 1st employed to evaluate associations between baseline characteristics and all-cause mortality. After the Schoenfeld residual test, variables with *P* < .05 in univariate analysis were subjected to LASSO regression and elastic network analysis, and then covariates were selected based on literature reports. To explore potential nonlinear relationships, restrictive cubic spline (RCS) regression was applied to model the association between baseline NPAR (continuous) and mortality, adjusting for multivariate confounders. Moreover, to determine the prognostic value of NPAR on all-cause mortality at different IR risks, we analyzed participants in the IR high-risk group, IR intermediate-risk group, and IR low-risk group separately. Subgroup analyses were conducted by age, sex, race, and other baseline factors, with interaction terms tested to examine consistency in the NPAR–mortality association. When >1 subgroup was involved, Bonferroni testing was used for multiple adjustment. Missing continuous data were handled via multiple imputation. Sensitivity analysis was used to verify the stability of the results. A 2-sided *P* ≤ .05 defined statistical significance.

## 3. Results

### 3.1. General characteristics of participants

After inclusion and exclusion criteria, a total of 1187 people were included in this study. The baseline data of the overall population, *T*1, *T*2, and *T*3 groups are shown in Table 1. There were significant differences in age, race, glucose metabolism, chronic bronchitis, kidney disease, HbA1c, FPG, WBC, and Hb among the 3 groups (*P *< .05), while there were no significant differences in gender, marital status, education level, ratio of family income to poverty, hypertension, coronary heart disease, TyG level, smoking, BMI, and platelet count (*P *> .05).

**Table 1:** Baseline characteristics of different levels of neutrophil percentage-to-albumin ratio in stroke survivors.

Variables	Total (n = 1187)	*T*1 (n = 401)	*T*2 (n = 403)	*T*3 (n = 383)	Statistic	*P*
Gender, n (%)					χ^2^ = 0.81	.667
Male	580 (48.86)	203 (50.62)	195 (48.39)	182 (47.52)		
Female	607 (51.14)	198 (49.38)	208 (51.61)	201 (52.48)		
Age	66.66 ± 10.65	64.82 ± 10.16	66.65 ± 10.56	68.59 ± 10.91	*F* = 12.50	<.001
Race, n (%)					χ^2^ = 35.85	<.001
Mexican American	154 (12.97)	51 (12.72)	52 (12.90)	51 (13.32)		
Other Hispanic	64 (5.39)	16 (3.99)	31 (7.69)	17 (4.44)		
Non-Hispanic White	540 (45.49)	151 (37.66)	190 (47.15)	199 (51.96)		
Non-Hispanic Black	353 (29.74)	159 (39.65)	104 (25.81)	90 (23.50)		
Other race	76 (6.40)	24 (5.99)	26 (6.45)	26 (6.79)		
Marital status, n (%)					χ^2^ = 4.63	.327
Married	582 (49.53)	203 (51.13)	201 (50.38)	178 (46.97)		
Never married	89 (7.57)	33 (8.31)	33 (8.27)	23 (6.07)		
Widowed/divorced/separated	504 (42.89)	161 (40.55)	165 (41.35)	178 (46.97)		
Education level, n (%)					χ^2^ = 1.10	.894
<12th grade	465 (39.24)	159 (39.65)	159 (39.55)	147 (38.48)		
High school grade	317 (26.75)	111 (27.68)	101 (25.12)	105 (27.49)		
College graduate or above	403 (34.01)	131 (32.67)	142 (35.32)	130 (34.03)		
Ratio of family income to poverty, n (%)					χ^2^ = 1.94	.748
<1.3	466 (39.26)	158 (39.40)	153 (37.97)	155 (40.47)		
1.3–3.5	498 (41.95)	161 (40.15)	175 (43.42)	162 (42.30)		
>3.5	223 (18.79)	82 (20.45)	75 (18.61)	66 (17.23)		
Hypertension, n (%)					χ^2^ = 5.40	.067
Yes	927 (78.10)	302 (75.31)	311 (77.17)	314 (81.98)		
No	260 (21.90)	99 (24.69)	92 (22.83)	69 (18.02)		
Glucose metabolism n (%)					χ^2^ = 18.23	.001
DM group	479 (40.35)	152 (37.91)	144 (35.73)	183 (47.78)		
Pre-DM group	353 (29.74)	137 (34.16)	118 (29.28)	98 (25.59)		
NGR group	355 (29.91)	112 (27.93)	141 (34.99)	102 (26.63)		
Coronary heart disease, n (%)					χ^2^ = 3.68	.159
Yes	216 (18.20)	64 (15.96)	71 (17.62)	81 (21.15)		
No	971 (81.80)	337 (84.04)	332 (82.38)	302 (78.85)		
Chronic bronchitis					χ^2^ = 14.02	<.001
Yes	120 (10.15)	30 (7.52)	33 (8.25)	57 (14.88)		
No	1062 (89.85)	369 (92.48)	367 (91.75)	326 (85.12)		
Kidneys disease					χ^2^ = 6.15	.046
Yes	95 (9.35)	31 (8.81)	24 (6.98)	40 (12.50)		
No	921 (90.65)	321 (91.19)	320 (93.02)	280 (87.50)		
TyG					χ^2^ = 3.91	.418
<8.5	306 (25.78)	103 (25.69)	108 (26.80)	95 (24.80)		
8.5–9.0	373 (31.42)	139 (34.66)	120 (29.78)	114 (29.77)		
>9.0	508 (42.80)	159 (39.65)	175 (43.42)	174 (45.43)		
Smoking, n (%)					χ^2^ = 2.49	.647
Every day	247 (20.83)	82 (20.45)	84 (20.84)	81 (21.20)		
Some days	32 (2.70)	14 (3.49)	7 (1.74)	11 (2.88)		
Not at all	907 (76.48)	305 (76.06)	312 (77.42)	290 (75.92)		
BMI	29.28 (25.29–34.05)	28.99 (25.80–33.20)	29.19 (24.90–33.62)	30.00 (25.10–34.90)	χ^2^ = 2.28	.319
HbA1c (%)	5.80 (5.50–6.50)	5.90 (5.50–6.50)	5.80 (5.40–6.30)	5.90 (5.50–6.60)	χ^2^ = 8.01	.018
FPG (mmol/L)	5.56 (5.00–6.83)	5.44 (4.94–6.50)	5.50 (5.00–6.44)	5.78 (5.11–7.61)	χ^2^ = 17.47	<.001
TG (mmol/L)	1.56 (1.07–2.22)	1.58 (1.05–2.26)	1.53 (1.11–2.17)	1.56 (1.06–2.25)	χ^2^ = 0.10	.951
WBC (1000 cells/µL)	7.10 (5.90–8.60)	6.40 (5.40–7.90)	7.20 (5.90–8.40)	7.90 (6.60–9.40)	χ^2^ = 80.60	<.001
Hb (g/dL)	13.69 ± 1.64	13.89 ± 1.51	13.86 ± 1.61	13.30 ± 1.73	F = 16.13	<.001
Platelet count (1000 cells/µL)	233.00 (194.75–277.00)	233.00 (196.00–272.00)	235.00 (198.00–278.50)	235.00 (191.50–284.50)	χ^2^ = 1.80	.407

Data were presented as mean ± SD, median and interquartile range, or as n (%).

BMI = body mass index, DM = diabetes mellitus, FPG = fasting plasma glucose, Hb = hemoglobin, HbA1c = glycosylated hemoglobin, PLT = platelet count, TG = triglycerides, TyG = triglyceride-glucose index, WBC = white blood cell count.

### 3.2. Results of covariate screening

Covariates were preliminarily screened by Cox univariate analysis, and the specific results were shown in Table 2. Figure S1, Supplemental Digital Content 1 shows the LASSO coefficient path plot, cross-validation plot, and variable screening coefficient plot. The parameters for elastic-net regression were set as follows: λ = 0.05, α = 0.5, area under the curve = 0.92. After the above screening methods, gender, age, race, marital status, education level, BMI, heart failure, kidney disease, WBC, and Hb were included as covariates.

**Table 2:** Cox univariate analysis of all-cause mortality in stroke survivors.

Variables	*z*	*P*	HR
Gender			
Male			1.00 (reference)
Female	−4.03	<.001	0.69 (0.58–0.83)
Age	13.52	<.001	1.08 (1.06–1.09)
Race			
Mexican American			1.00 (reference)
Other Hispanic	0.15	.882	1.04 (0.62–1.73)
Non-Hispanic White	3.4	<.001	1.60 (1.22–2.09)
Non-Hispanic Black	−0.04	.971	0.99 (0.74–1.34)
Other race	−0.31	.754	0.92 (0.55–1.54)
Marital status			
Married			1.00 (reference)
Never married	−1.39	.164	0.75 (0.50–1.12)
Widowed/divorced/separated	1.99	.046	1.20 (1.01–1.45)
Education level			
<12th graduate			1.00 (reference)
High School graduate	−0.07	.945	0.99 (0.80–1.23)
College graduate or above	−2.41	.016	0.77 (0.62–0.95)
Ratio of family income to poverty			1.00 (reference)
<1.3	0.9	.37	1.09 (0.90–1.33)
1.3–3.5	-1.99	.046	0.77 (0.60–0.99)
>3.5			
Hypertension	1.87	.062	1.24 (0.99–1.54)
Glucose metabolism			
DM group			1.00 (reference)
Pre-DM group	−1.8	.071	0.82 (0.66–1.02)
NGR group	−3.31	<.001	0.70 (0.57–0.86)
Coronary heart disease	3.82	<.001	1.51 (1.22–1.86)
Heart failure	6.74	<.001	2.04 (1.66–2.51)
Chronic bronchitis	0.42	.675	1.06 (0.80–1.42)
Kidneys disease	2.71	.007	1.51 (1.12–2.02)
TyG			
<8.5			1.00 (reference)
8.5–9.0	0.25	.8	1.03 (0.81–1.31)
>9.0	0.43	.669	1.05 (0.84–1.31)
Smoking, n (%)			
Every day			1.00 (reference)
Some days	0.25	.806	1.08 (0.60–1.92)
Not at all	0.96	.338	1.12 (0.89–1.40)
BMI	−3.04	.002	0.98 (0.96–0.99)
HbA1c (%)	1.64	.1	1.05 (0.99–1.11)
FPG (mmol/L)	2.18	.029	1.03 (1.01–1.06)
TG (mmol/L)	−0.55	.583	0.98 (0.90–1.06)
WBC (1000 cells/µL)	3.52	<.001	1.07 (1.03–1.11)
Hb (g/dL)	−3.42	<.001	0.91 (0.86–0.96)
PLT (1000 cells/µL)	−3.17	.002	0.99 (0.99–0.99)

BMI = body mass index, DM = diabetes mellitus, FPG = fasting plasma glucose, Hb = hemoglobin, HbA1c = glycosylated hemoglobin, HR = hazard ratio, PLT = platelet count, TG = triglycerides, TyG = triglyceride-glucose index, WBC = white blood cell count.

### 3.3. Predictive value of NPAR for all-cause mortality in participants

During a median follow-up of 86 months, 496 participants died, with a median survival time of 133 months and an overall mortality rate of 41.79%. KM survival curves showed progressively increasing mortality risk across *T*1 to *T*3 groups, with a statistically significant difference (*P* < .001) (Fig. 2A). Cox proportional‑hazards models confirmed a significant association between baseline NPAR levels and mortality. Baseline NPAR was entered into Cox regression analyses both as a continuous variable and as a categorical variable defined by tertiles. After adjustment for all covariates (model 3), each 1‑unit increment in baseline NPAR was associated with a 10% higher mortality risk (hazard ratio [HR] = 1.10, 95% confidence interval [CI]: 1.07–1.14). Likewise, in model 3, mortality risk rose gradually across NPAR tertiles: participants in the highest tertile (*T*3) had a 75% higher mortality risk relative to those in the lowest tertile (*T*1) (HR = 1.68, 95% CI: 1.33–2.14). Detailed results are presented in Table 3.

**Table 3:** Cox multivariate analysis of all-cause mortality among stroke survivors in different models.

Group	Model 1		Model 2		Model 3	
	*P*	HR (95% CI)	*P*	HR (95% CI)	*P*	HR (95% CI)
NPAR (continuous variables)	<.001	1.15 (1.12–1.19)	<.001	1.13 (1.10–1.17)	<.001	1.10 (1.07–1.14)
NPAR (categorical variables)						
*T*1		1.00 (reference)		1.00 (reference)		1.00 (reference)
*T*2	.013	1.33 (1.06–1.67)	.04	1.28 (1.01–1.61)	.223	1.16 (0.91–1.47)
*T*3	<.001	2.16 (1.73–2.70)	<.001	1.95 (1.55–2.45)	<.001	1.68 (1.33–2.14)

Model 1: Unadjusted.

Model 2: Adjusted gender, age, race, marital status, education level and BMI.

Model 3: Model 2 + adjusted heart failure, kidney disease, WBC and Hb.

BMI = body mass index, CI = confidence interval, Hb = hemoglobin, HR = hazard ratio, NAPR = neutrophil percentage-to-albumin ratio, WBC = white blood cell count.

**Figure 2.**
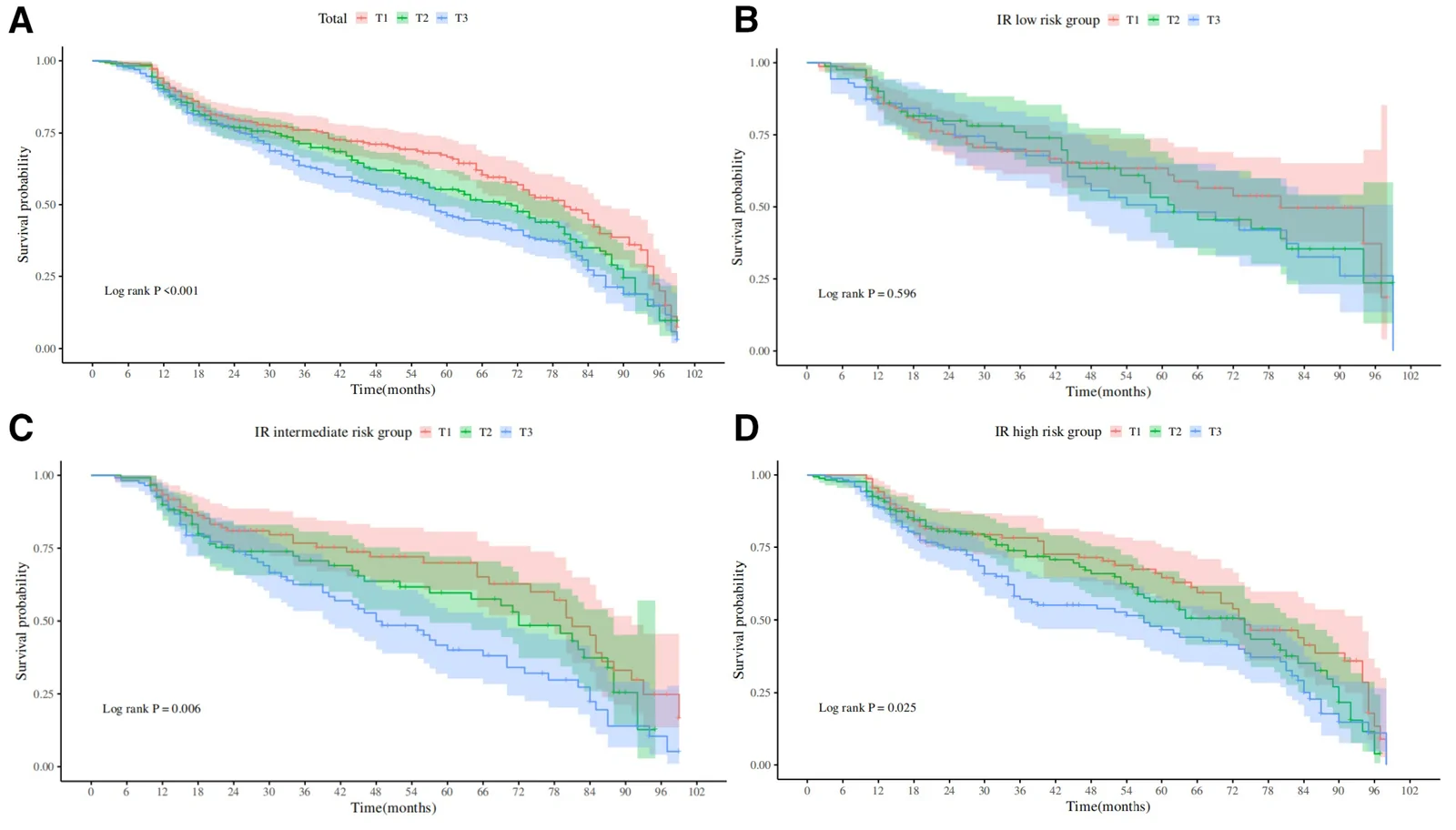
The Kaplan–Meier analysis for mortality rate was based on NPAR tertiles for (A) total participants, (B) participants with IR low-risk, (C) participants with IR intermediate-risk, and (D) participants with high- risk. IR = insulin resistance, NPAR = neutrophil percentage-to-albumin ratio.

### 3.4. The association between NPAR at different TyG levels and all-cause mortality

During a median follow-up period, deaths occurred in 116 of 306 participants (37.9%) in the IR low-risk group, 158 of 373 participants (42.4%) in the IR intermediate-risk group, and 222 of 508 participants (43.7%) in the IR high-risk group. KM survival curves, stratified by NPAR tertiles, revealed a statistically significant gradient in mortality risk among participants in the IR intermediate-risk and high-risk groups (log-rank *P* < .001 for both). In contrast, no significant difference in survival was observed across NPAR tertiles within the IR low-risk group (log-rank *P* = .088). The corresponding survival curves are presented in Figure 2B–D.

When modeled as a continuous variable in the fully adjusted model (model 3), NPAR was significantly associated with all-cause mortality in the IR intermediate-risk group (HR = 1.18, 95% CI: 1.10–1.26) and the IR high-risk group (HR = 1.10, 95% CI: 1.05–1.16). No significant association was detected in the IR low-risk group (*P* = .061). In the categorical analysis based on NPAR tertiles, participants in the highest tertile (*T*3) had a significantly higher risk of all-cause mortality relative to those in the lowest tertile (*T*1) across all adjustment models in both the IR high-risk and intermediate-risk groups. In contrast, for the IR low-risk group, the significant association was only observed in the minimally adjusted model (model 1) and diminished to non-significance after comprehensive adjustment for potential confounders. Detailed results are presented in Table 4.

**Table 4:** Association between neutrophil percentage-to-albumin ratio and all-cause mortality according to IR risk.

Group	Model 1		Model 2		Model 3	
	*P*	HR (95% CI)	*P*	HR (95% CI)	*P*	HR (95% CI)
TyG <8.5						
NPAR (continuous variables)	<.001	1.13 (1.06–1.20)	.001	1.12 (1.05–1.20)	.061	1.08 (1.00–1.17)
NPAR (categorical variables)						
*T*1	1.00 (reference)		1.00 (reference)		1.00 (reference)	
*T*2	.095	1.47 (0.94–2.31)	.336	1.27 (0.78–2.06)	.573	1.15 (0.71–1.87)
*T*3	.035	1.67 (1.04–2.70)	.235	1.37 (0.82–2.29)	.754	1.09 (0.63–1.90)
8.5 ≤ TyG ≤ 9.0						
NPAR (continuous variables)	<.001	1.19 (1.13–1.27)	<.001	1.20 (1.12–1.28)	<.001	1.18 (1.10–1.26)
NPAR (categorical variables)						
*T*1	1.00 (reference)		1.00 (reference)		1.00 (reference)	
*T*2	.359	1.21 (0.81–1.82)	.66	1.10 (0.71–1.71)	.819	1.05 (0.63–1.52)
*T*3	<.001	2.96 (2.02–4.36)	<.001	2.58 (1.68–3.96)	<.001	2.43 (1.57–3.75)
TyG >9.0						
NPAR (continuous variables)	<.001	1.13 (1.08–1.19)	<.001	1.13 (1.07–1.18)	<.001	1.10 (1.05–1.16)
NPAR (categorical variables)						
*T*1	1.00 (reference)		1.00 (reference)		1.00 (reference)	
*T*2	.108	1.32 (0.94–1.85)	.057	1.40 (0.99–1.98)	.153	1.30 (0.91–1.85)
*T*3	<.001	1.94 (1.40–2.70)	<.001	1.96 (1.40–2.76)	.003	1.69 (1.19–2.40)

Model 1: Unadjusted.

Model 2: Adjusted gender, age, race, marital status, education level and BMI.

Model 3: Model 2 + adjusted heart failure, kidney disease, WBC and Hb.

BMI = body mass index, CI = confidence interval, Hb = hemoglobin, HR = hazard ratio, IR = insulin resistance, NAPR = neutrophil percentage-to-albumin ratio, TyG = triglyceride-glucose index, WBC = white blood cell count.

### 3.5. RCS curves of the relationship between NPAR and mortality

After adjusting for covariates, RCS curve analysis was performed for all participants and for subgroups stratified by IR status, including the low-risk, intermediate-risk, and high-risk groups. Linear associations between NPAR and all-cause mortality were observed in the overall cohort (*P* for overall <.001, *P* for nonlinear = .291), the IR intermediate‑risk group (*P* for overall <.001, *P* for nonlinear = .088), and the IR high‑risk group (*P* for overall < .001, *P* for nonlinear = .236). In contrast, no significant association was identified in the IR low‑risk group (*P* for overall = .144, *P* for nonlinear = .386). Detailed RCS results are displayed in Figure 3.

**Figure 3.**
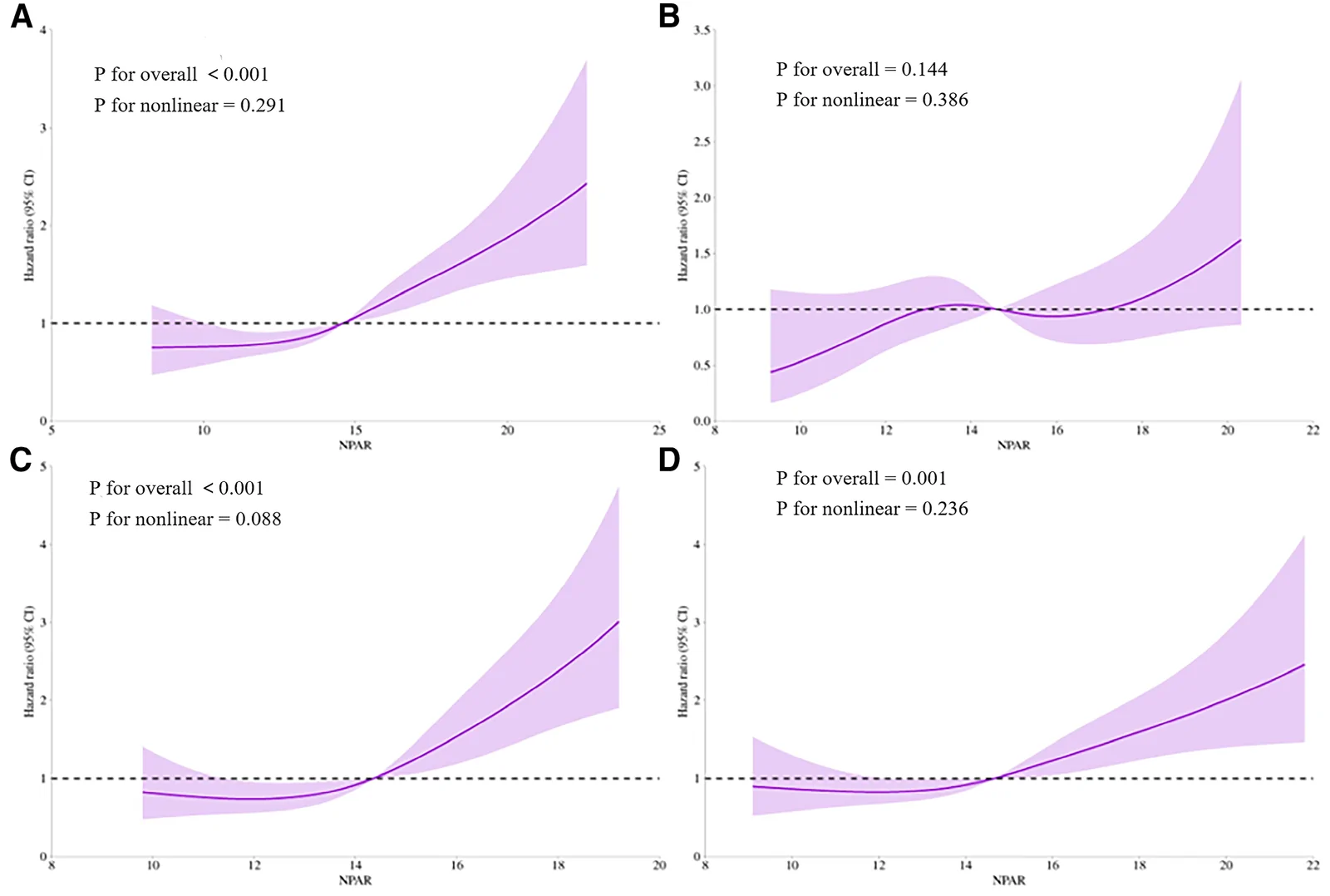
Association of NPAR and the risk of mortality rate using a multivariable-adjusted restricted cubic splines model. Restricted cubic spline analysis has 4 knots at the 5th, 35th, 65th, and 95th percentiles of NPAR. (A) Total participants; (B) participants with IR low-risk; (C) participants with IR intermediate-risk; and (D) participants with IR high-risk group. CI = confidence interval, IR = insulin resistance, NPAR = neutrophil percentage-to-albumin ratio.

### 3.6. Subgroup analysis

Subgroup analyses were conducted to evaluate the association between NPAR and all-cause mortality across demographic and clinical subgroups. Age was stratified at 60 years, and BMI was stratified at 25 kg/m^2^. No significant interactive effects were observed in most subgroups, supporting the robustness of the NPAR–mortality association across gender, glucose metabolism status, heart failure status, and BMI. Notably, a significant interaction was detected across TyG-defined IR subgroups (*P* for interaction = .047). Given the 3 IR subgroups, the Bonferroni-corrected significance threshold was set at *P* < .017. Stratified analyses further demonstrated that the predictive association of NPAR with mortality was significant in the IR intermediate-risk (*P* < .001) and IR high-risk groups (*P* < .001) but nonsignificant in the IR low-risk group (*P *= .136). Detailed subgroup analysis results are presented in Table 5.

**Table 5:** Subgroup and interaction analyses of the association between neutrophil percentage-to-albumin ratio and total participant.

Variables	n (%)	HR (95% CI)	*P*	*P* for interaction
All patients	1187 (100.00)	1.15 (1.12–1.19)	<.001	
Gender				.300
Male	580 (48.86)	1.10 (1.05–1.15)	<.001	
Female	607 (51.14)	1.12 (1.06–1.19)	<.001	
Age				.732
≤60	319 (26.87)	1.12 (1.00–1.26)	.041	
>60	868 (73.13)	1.10 (1.06–1.14)	<.001	
Race, n (%)				.116
Mexican American	154 (12.97)	1.24 (1.10–1.41)	<.001	
Other Hispanic	64 (5.39)	1.28 (1.02–1.62)	.036	
Non-Hispanic White	540 (45.49)	1.09 (1.04–1.15)	<.001	
Non-Hispanic Black	353 (29.74)	1.08 (1.01–1.15)	.022	
Other race	76 (6.40)	0.95 (0.77–1.17)	.614	
Hypertension				.846
Yes	927 (78.10)	1.11 (1.07–1.16)	<.001	
No	260 (21.90)	1.05 (0.96–1.14)	.304	
Glucose metabolism				.473
DM group	479 (40.35)	1.10 (1.05–1.16)	<.001	
Pre-DM group	353 (29.74)	1.10 (1.02–1.18)	.014	
NGR group	355 (29.91)	1.13 (1.05–1.22)	<.001	
Coronary heart disease				.473
Yes	216 (18.20)	1.04 (0.97–1.13)	.256	
No	971 (81.80)	1.12 (1.07–1.16)	<.001	
Heart failure				.912
Yes	208 (17.70)	1.11 (1.03–1.19)	.005	
No	967 (82.30)	1.10 (1.06–1.15)	<.001	
Chronic bronchitis				.431
Yes	120 (10.15)	1.09 (0.98–1.21)	.112	
No	1062 (89.85)	1.11 (1.07–1.15)	<.001	
Kidneys disease				.073
Yes	95 (9.35)	1.28 (1.13–1.45)	<.001	
No	921 (90.65)	1.09 (1.05–1.13)	<.001	
TyG				.047
<8.5	306 (25.78)	1.09 (0.94–1.18)	.136	
8.5–9.0	373 (31.42)	1.18 (1.10–1.26)	<.001	
>9.0	508 (42.80)	1.10 (1.05–1.16)	<.001	
BMI				.145
≤25	280 (23.59)	1.11 (1.02–1.21)	.013	
>25	907 (76.41)	1.10 (1.05–1.14)	<.001	

Adjusted for gender, age, race, marital status, education level, BMI, heart failure, kidney disease, WBC, and Hb.

BMI = body mass index, CI = confidence interval, DM = diabetes mellitus, Hb = hemoglobin, HR = hazard ratio, NGR = normal glucose regulation, TyG = triglyceride-glucose index, WBC = white blood cell count.

### 3.7. Sensitivity analysis

Sensitivity analyses were performed to verify the robustness of the primary findings. After excluding the top and bottom 5% of the NPAR distribution, Cox proportional‑hazards models were reperformed across TyG subgroups with multivariable adjustment, and the overall results remained consistent (Table S1, Supplemental Digital Content 2). Further sensitivity analysis excluding participants with missing continuous variable data (n = 167) yielded materially unchanged associations between baseline NPAR and all‑cause mortality across IR strata (Table S2, Supplemental Digital Content 3).

## 4. Discussion

This prospective observational cohort study utilized data from 1187 stroke survivors in the NHANES database from 1999 to 2018 to investigate the prognostic value of the neutrophil NPAR. Using multivariate Cox proportional hazards regression models adjusted for demographics and relevant covariates, we identified NPAR as an an independent risk factor for all-cause mortality, whether analyzed as a continuous or categorical variable. Notably, this association varied significantly across strata of IR defined by the TyG. Specifically, the positive association between NPAR and mortality was more pronounced in the IR intermediate-risk and IR high-risk groups but was not observed in the IR low-risk group. Subgroup analyses further confirmed that the risk associated with NPAR differed significantly by TyG level. To the best of our knowledge, this is the 1st study to report a differential effect of TyG strata on the NPAR–mortality relationship in stroke survivors. These findings suggest that the association between NPAR and mortality risk in stroke survivors may be modified by IR status. The biological mechanisms underlying this potential effect modification remain speculative and require validation through mechanistic studies. NPAR is a novel inflammatory-nutritional marker that integrates neutrophil and albumin levels. Its association with mortality is stronger among individuals with intermediate or high IR risk, highlighting the importance of metabolic–inflammatory interactions in secondary stroke prevention. These findings provide preliminary evidence for a potential risk stratification strategy incorporating NPAR and IR status to aid in prognostic assessment, suggesting a possible role for individualized risk evaluation in stroke survivors.

TyG has gained acceptance as a practical surrogate marker of insulin sensitivity since its introduction by Simental-Mendía et al in healthy populations.^[9]^ In line with prior reports, IR was defined as a TyG exceeding 8.5.^[10,11]^ TyG, especially at a threshold around 9, represents a strong integrated indicator of metabolic risk, whereby the severity of IR is associated with increased mortality from related diseases.^[12]^ Based on previous studies on the optimal threshold of the TyG index, we divided the TyG index into 3 groups: <8.5, 8.5–9, and >9. In addition, it has been proved that TyG-related composite indicators are associated with poststroke mortality, and stroke patients with reduced TyG-BMI and increased TyG-waist-to-height ratio levels often face a higher risk of death.^[13]^ Qian et al demonstrated that the chain-mediated effects of atherogenic index of plasma and TyG significantly increased the risk of poststroke mortality in an analysis of the China Health and Retirement Longitudinal Study database.^[14]^ This finding highlights the complex interplay between lipid and glucose metabolism in stroke prognosis and offers a novel perspective on metabolic management after stroke.

As a new inflammatory index, NPAR is calculated by detecting the percentage of neutrophils and albumin content, and the detection method is simple and easy to obtain. Neutrophil percentage is an objective quantitative indicator of inflammatory response, and albumin is an effective measure of nutritional status. The ratio of the 2 has been shown to be associated with poor prognosis of a variety of diseases, including sepsis, cancer, liver cirrhosis, diabetes, chest pain, and so on.^[15–19]^ Moreover, NPAR may also be a predictor of diseases, including depression, periodontitis, breast cancer, etc.^[20–22]^ However, these conclusions are mainly based on cross-sectional studies and lack strong causal associations.

NPAR is also closely related to the occurrence and prognosis of stroke. Xu et al^[23]^ analyzed the association between NPAR (albumin in g/dL) and stroke and found that there was a nonlinear positive correlation between NPAR and stroke: The threshold effect analysis suggested that the inflection point was NPAR = 13.81, with odds ratios before and after the inflection point of 1.13 and 1.04, respectively, implying that there was always a positive association between NPAR and stroke, and that the increasing trend of stroke risk began to slow down as the level of NPAR increased. In addition, NPAR (albumin in g/L) was independently associated with the risk of recurrent acute ischemic stroke within 3 months (odds ratio 9.71, 95% CI: 3.05–31.62, *P* < .001), the best cutoff value was 1.78, the sensitivity was 0.80, the specificity was 0.75, and the maximum area under the curve was 0.78, suggesting that NPAR was a powerful predictor of recurrence at 3 months after the 1st stroke.^[24]^ Zhang et al utilized the NHANES database to analyze 6 inflammatory biomarkers and identified NPAR and systemic inflammation response index (SIRI) as key prognostic markers for stroke through machine learning approaches.^[25]^ Using consensus clustering based on SIRI and NPAR levels, they stratified patients into 2 distinct inflammatory subgroups with differing mortality risks. Their findings suggest that enhanced monitoring and management of patients with elevated NPAR and SIRI levels may improve survival outcomes. However, their study did not account for the potential confounding effects of wasting diseases. Another study, analyzing clinical data from 940 subjects in the Medical Information Mart for Intensive Care database, demonstrated that after adjusting for confounding factors, elevated NPAR levels were strongly associated with an increased risk of mortality in stroke patients, irrespective of whether the endpoint was 30-, 90-, or 365-day mortality.^[26]^

Our results showed that in the overall cohort, with NPAR as the key factor, the risk of death was 75% higher in the *T*3 group than in the *T*1 group after adjusting for all covariates, and a 1-unit increase in NPAR was associated with a 10% higher risk of death, which is similar to previous studies. We grouped patients according to TyG level. After adjusting all covariates, the association between NAPR and mortality was not the same: in the IR intermediate-risk group, *T*3 had a 143% increased risk of death compared with *T*1, and for each 1-unit increase in NPAR, the risk of death increased by 18%; in the IR high-risk group, the risk of death increased by 69% in *T*3 compared with *T*1, and the risk of death increased by 10% for each 1-unit increase in NPAR. NPAR was not associated with mortality in the IR low-risk group as either a continuous variable or a categorical variable. The dose-response relationship between NPAR and mortality was analyzed by RCS curves. Linear associations between NPAR and mortality were observed in the overall participants, as well as in the IR intermediate and high-risk groups, whereas no such association was identified in the IR low-risk group. Interaction analyses demonstrated that the association between NPAR and mortality was significantly modified by TyG level with a stronger effect observed in the IR intermediate- and high-risk groups compared with the IR low-risk group. The absence of significant interactions in other subgroups further supports the stability of this association. Subgroup analysis indicated an interaction between NPAR and mortality across TyG subgroups. Multiple risk testing was performed because of the presence of 3 subgroups. After Bonferroni correction, significant differences were observed in the IR intermediate- and high-risk groups compared with the low-risk group, suggesting that NPAR is more strongly associated with survival time in the IR intermediate- and high-risk populations than in the low-risk group.

Notably, univariate Cox regression analysis showed that TyG levels were not significantly associated with mortality, indicating that the stratum‑specific association between NPAR and mortality was not directly mediated by TyG values alone. Although the modifying effect of TyG status on the NPAR–mortality relationship was statistically evident, the underlying biological mechanism remains speculative in this observational cohort. We hypothesize that lower TyG status may confer a marginal protective effect against inflammation‑related damage under elevated NPAR conditions, whereas higher TyG levels may attenuate such benefits. Nevertheless, this mechanistic interpretation requires further prospective and experimental validation.

### 4.1. Limitations

This large-scale population-based investigation examined the relationship between NPAR and mortality among stroke survivors. The study utilized high-quality, nationally representative longitudinal data from a US cohort encompassing adults aged 40 to 80 years across diverse geographic regions. To ensure robust findings, we adjusted for potential confounding factors that could bias the observed associations. Over a maximum follow-up period of approximately 20 years, our analysis revealed a TyG level-dependent association between NPAR and mortality: the strength of this association exhibited a positive gradient with increasing TyG levels.

This study has several limitations: recall bias: some of the data were obtained through questionnaire surveys, which may lead to recall bias, including reliance on the patients’ self-reported doctor’s diagnosis results. However, the use of standardized interviewer-administered questionnaires in NHANES, along with the inclusion of objectively measured biomarkers (e.g., HbA1c and blood cell counts) for key exposures, partially mitigates this concern. Future studies incorporating medical record validation or biomarker-confirmed exposures would strengthen causal inference. Unmeasured confounders: despite adjusting for potential confounders, key variables such as stroke onset age, functional independence, rehabilitation adherence, were unavailable or insufficient in the database, possibly introducing residual confounding. Single NPAR measurement: NPAR was assessed only once, preventing evaluation of its dynamic changes. Although we excluded patients with wasting diseases, acute infectious conditions at the time of measurement could still influence NPAR levels. stroke subtype uncertainty: the database lacked differentiation between ischemic and hemorrhagic stroke subtypes. survey weights: we did not take into account the survey weights, which may have prevented the results from being generalizable to the entire NHANES population. While survey weights are essential for prevalence estimation in NHANES, their application in survival analysis with complex covariate adjustment remains debated. Not using survey weights is generally considered acceptable in etiologic studies when the primary aim is to estimate association rather than population representative rates.

## 5. Conclusions

In summary, this study observed a significant association between NPAR and mortality in stroke survivors, with higher NPAR levels corresponding to a higher risk of death. The association varied by TyG level, appearing strongest in the IR intermediate-risk group, followed by the IR high-risk group, with no significant association detected in the IR low-risk group. Subgroup and sensitivity analyses indicated that these findings were generally robust. Taken together, these results highlight the potential value of NPAR as a risk stratification marker in stroke populations and suggest that considering TyG levels may inform more individualized risk assessment. However, given the observational design, further prospective studies are needed to validate these findings before clinical applications can be considered.

## Acknowledgments

We would like to express our gratitude to the NHANES database for providing its platform and to the contributors who have shared their valuable datasets. We also extend our sincere thanks to all the participants who took part in this study.

## Author contributions

**Funding acquisition:** Sheng Zhang.

**Writing – original draft:** Ning Liu.

**Writing – review & editing:** Zhongzhou Su, Sheng Zhang.






