# Supplementary material for: Association between neutrophil percentage-to-albumin ratio and all-cause mortality in stroke survivors stratified by triglyceride-glucose index: A prospective observational study based on the NHANES database from 1999 to 2018
Source: Medicine (Baltimore). 2026 Oct 9;105(41):e50968. doi: 10.1097/MD.0000000000050968 (PMC13657450; doi:10.1097/MD.0000000000050968)
Supplement: Supplementary file 1 [file medi-105-e50968-s001.pdf]

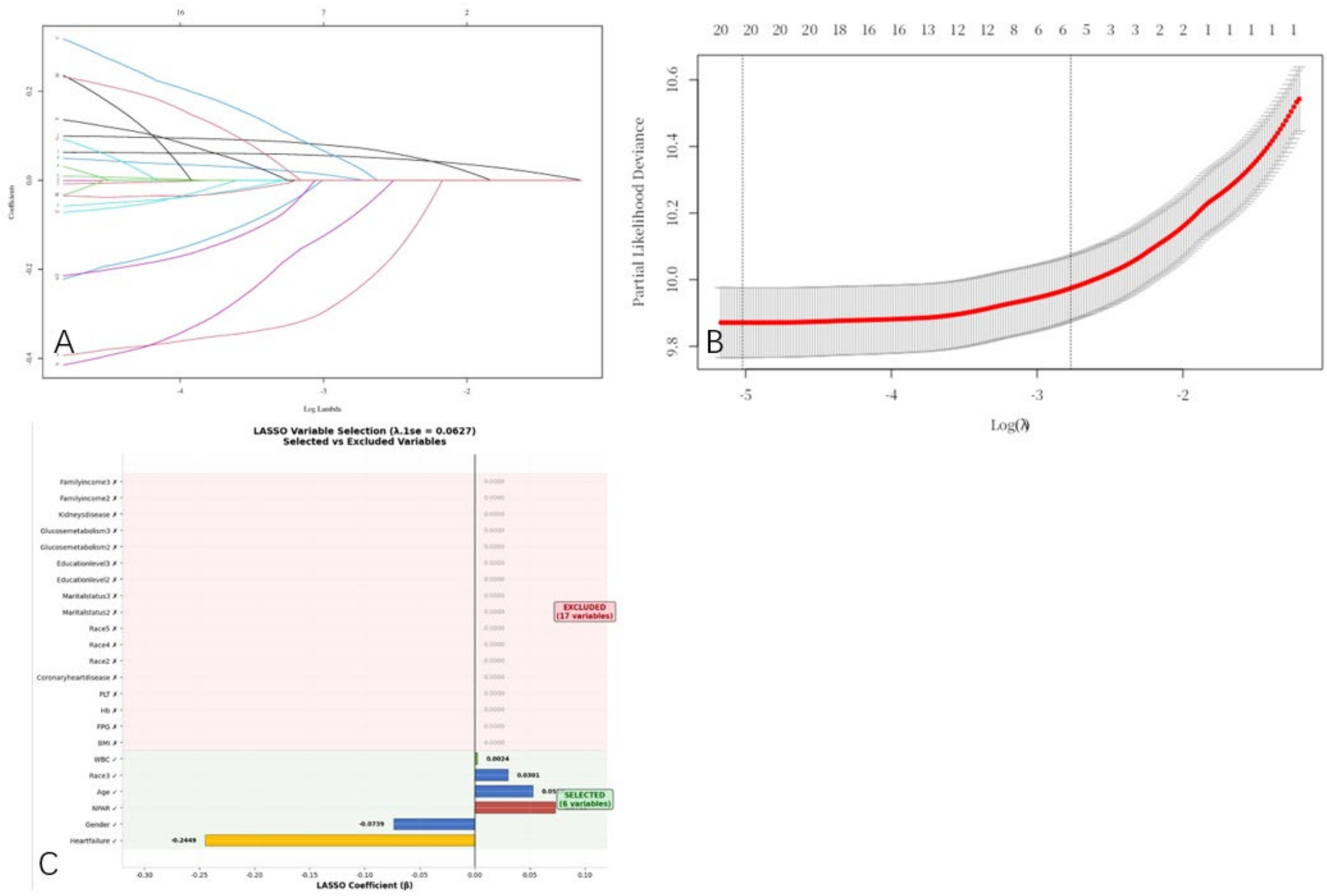

Figure S1: Description of covariate screening. A. LASSO regression was performed based on the variables preliminarily screened by univariate COX regression. B. Cross-validation plot of LASSO regression. C. Coefficient plot of screening variables
