# Supplementary material for: Association between neutrophil percentage-to-albumin ratio and all-cause mortality in stroke survivors stratified by triglyceride-glucose index: A prospective observational study based on the NHANES database from 1999 to 2018
Source: Medicine (Baltimore). 2026 Oct 9;105(41):e50968. doi: 10.1097/MD.0000000000050968 (PMC13657450; doi:10.1097/MD.0000000000050968)
Supplement: Supplementary file 2 [file medi-105-e50968-s002.pdf]

Table S1: Sensitivity analysis with 5% extreme values before and after removal

Group	Model 1		Model 2		Model3	
	p	HR（95%CI）	p	HR（95%CI）	p	HR（95%CI）
TyG＜8.5						
NPAR(continuous variables)	0.047	1.10 (1.01 ~ 1.21	0.169	1.08 (0.97 ~ 1.19)	0.312	1.06 (0.95 ~ 1.18)
NPAR(categorical variables)						
T1		1.00 (Reference)		1.00 (Reference)		1.00 (Reference)
T2	0.334	1.27 (0.78 ~ 2.05)	0.705	0.91 (0.55 ~ 1.51)	0.459	0.82 (0.49 ~ 1.38)
T3	0.165	1.42 (0.86 ~ 2.34)	0.814	1.07 (0.63 ~ 1.81)	0.957	1.02 (0.59 ~ 1.76)
8.5≤TyG≤9.0						
NPAR(continuous variables)	<.001	1.22 (1.13 ~ 1.32)	<.001	1.21 (1.11 ~ 1.32)	<.001	1.21 (1.10 ~ 1.32)
NPAR(categorical variables)						
T1		1.00 (Reference)		1.00 (Reference)		1.00 (Reference)
T2	0.321	1.24 (0.81 ~ 1.90)	0.464	1.18 (0.75 ~ 1.86)	0.980	1.01 (0.63 ~ 1.62)
T3	<.001	2.73 (1.83 ~ 4.06)	<.001	2.56 (1.64 ~ 3.99)	<.001	2.45 (1.55 ~ 3.87)
TyG＞9.0						
NPAR(continuous variables)	<.001	1.15 (1.07 ~ 1.23)	<.001	1.15 (1.07 ~ 1.24)	0.001	1.13 (1.05 ~ 1.22)

NPAR(categorical variables)						
T1		1.00 (Reference)		1.00 (Reference)		1.00 (Reference)
T2	0.494	1.13 (0.79 ~ 1.62)	0.276	1.23 (0.85 ~ 1.77)	0.535	1.13 (0.77 ~ 1.64)
T3	0.002	1.73 (1.23 ~ 2.44)	0.001	1.81 (1.27 ~ 2.58)	0.009	1.63 (1.13 ~ 2.34)

Model 1: unadjusted

Model 2: adjusted gender, age , race, marital status, education level and BMI

Model 3: model2+adjusted heart failure, kidney disease, WBC and Hb

NAPR：Neutrophil percentage-to-albumin ratio；BMI：Body Mass Index；WBC: White blood cell count；Hb: Hemoglobin
