# Supplementary material for: Association between neutrophil percentage-to-albumin ratio and all-cause mortality in stroke survivors stratified by triglyceride-glucose index: A prospective observational study based on the NHANES database from 1999 to 2018
Source: Medicine (Baltimore). 2026 Oct 9;105(41):e50968. doi: 10.1097/MD.0000000000050968 (PMC13657450; doi:10.1097/MD.0000000000050968)
Supplement: Supplementary file 3 [file medi-105-e50968-s003.pdf]

Table S2: Sensitivity analysis with missing values excluded

Group	Model 1		Model 2		Model3	
	p	HR（95%CI）	p	HR（95%CI）	p	HR（95%CI）
TyG＜8.5						
NPAR(continuous variables)	< .001	1.14 (1.07 ~ 1.22)	< .001	1.14 (1.06 ~ 1.23)	0.075	1.08 (0.99 ~ 1.18)
NPAR(categorical variables)						
T1		1.00 (Reference)		1.00 (Reference)		1.00 (Reference)
T2	0.09	1.55 (0.93 ~ 2.58)	0.278	1.39 (0.80 ~ 2.41)	0.460	1.28 (0.74 ~ 2.22)
T3	0.02	1.86 (1.10 ~ 3.13)	0.104	1.58 (0.90 ~ 2.77)	0.542	1.20 (0.65 ~ 2.23)
8.5≤TyG≤9.0						
NPAR(continuous variables)	<.001	1.18 (1.10 ~ 1.26)	<.001	1.16 (1.07 ~ 1.26)	0.001	1.16 (1.06 ~ 1.26)
NPAR(categorical variables)						
T1		1.00 (Reference)		1.00 (Reference)		1.00 (Reference)
T2	0.925	0.98 (0.62 ~ 1.54)	0.788	0.93 (0.57 ~ 1.53)	0.411	0.81 (0.48 ~ 1.35)
T3	<.001	2.68 (1.75 ~ 4.09)	0.004	2.06 (1.28 ~ 3.31)	0.007	1.99 (1.21 ~ 3.26)
TyG＞9.0						
NPAR(continuous variables)	<.001	1.10 (1.04 ~ 1.17)	0.006	1.09 (1.02 ~ 1.16)	0.042	1.05 (1.01 ~ 1.13)
NPAR(categorical variables)						

T1		1.00 (Reference)		1.00 (Reference)		1.00 (Reference)
T2	0.33	1.20 (0.83 ~ 1.71)	0.383	1.18 (0.81 ~ 1.70)	0.681	1.09 (0.72 ~ 1.65)
T3	0.014	1.58 (1.10 ~ 2.28)	0.029	1.53 (1.05 ~ 2.24)	0.048	1.26 (1.03 ~ 2.06)

Model 1: unadjusted

Model 2: adjusted gender, age , race, marital status, education level and BMI

Model 3: model2+adjusted heart failure, kidney disease, WBC and Hb

NAPR：Neutrophil percentage-to-albumin ratio；BMI：Body Mass Index；WBC: White blood cell count；Hb: Hemoglobin
